# Formation of self-nitrogen-doping activated carbon from Fish/sawdust/ZnCl_2_ by hydrothermal and pyrolysis for toxic chromium adsorption from wastewater

**DOI:** 10.1038/s41598-023-38697-3

**Published:** 2023-07-18

**Authors:** Mohamed A. El-Nemr, Mohamed A. Hassaan, Ibrahim Ashour

**Affiliations:** 1 Department of Chemical Engineering, Faculty of Engineering, Minia University, Minia, 61519 Egypt; 2 Environment Division, National Institute of Oceanography and Fisheries (NIOF), Kayet Bey, El-Anfoushy, Alexandria, Egypt

**Keywords:** Environmental sciences, Environmental chemistry

## Abstract

This study gives a description of the formation of self-nitrogen doped activated carbon (NDAC) by a novel way of employing fish meal (mixture of *Atherina hepseetus* and *Sardina pilchardus* of 60% protein) as nitrogen dopant, ZnCl_2_ as impregnate agent, sawdust as carbon source and water with a mass ratio (2:1:1:12), which subjected to the hydrothermal process. The hydrothermal mixture was oven dried and carbonized under a flow of nitrogen for one h at 600, 700, and 800 °C. The characterization of NDAC was performed by using various analytical techniques analyses. The synthesized NDAC exhibited unique features such as microporous structure (1.84 ~ 2.01 nm), high surface area (437.51 ~ 680.86 m^2^/g), the volume of total pores (0.22 ~ 0.32 cm^3^/g) and nitrogen content (12.82 ~ 13.73%). Batch removal tests were achieved to investigate the impact of chromium ions starting concentration (100–400 mg/L), NDAC dose (0.5–2.5 g/L), pH and contact time (5–120 min). Such helpful characteristics of NDAC, particularly for NDAC600, were suitable to use as an excellent adsorbent for Cr^6+^ ions with a maximum adsorption capacity (*Q*_m_) (769.23 mg/g), and the highest chromium ions adsorption uptake (81.18%) was obtained at pH value 1.5 at room temperature. Both Halsey and Temkin models fitted the adsorption data quite reasonably. The uptake of toxic chromium ions is best represented with pseudo-second-order rate kinetics data.

## Introduction

Water is life, a natural resource essential for the survival and growth of living organisms. Water is strongly needed to meet the basic demands of a population, social and economic ambitions, agriculture, urbanization, industrialization and many other uses^1^. Pollution of water, air and land by toxic metal ions in overcrowded urban areas due to the rapid expansion of industrial activities and population enlargement became a global problem^2^. Recently, through the twentieth century, the requirement for pure water is becoming challenging, and the awareness to protect our environment from pollution has developed. In particular, the growth of contamination by inorganic micro-pollutants such as heavy metals attracted the concern of many researchers because they are persistent, very toxic and sometimes have a deadly effect^3^.

Heavy metals are, in many cases, toxic and cause degradation to plant and aquatic life as well as harm to the human being^4^. In recent decades, exposure to an environment contaminated with heavy metals has become a severe environmental risk across the globe^4^. Chromium is a naturally occurring element found during volcanic eruptions in dust, rocks, and soil. The EPA (US Environmental Protection Agency) categorized chromium as one of nature's most common toxic environmental pollutants^5^. Chromium and its compounds mainly result from diverse industrial activities such as the leather industry^6,7^. For instance, in India, the leather tanning industries processing caused a high influent of (2000 ~ 32,000 tons/year of Cr^6+^) on the environment^8,9^. Also, chromium is used extensively in electroplating, chromic acid, drilling muds, catalytic reagents and refractory steel^10^.

Many anthropogenic activities such as planting of metal, treatment of water in the cooling tower in various industries, wood conservation, pigment and electrical and electronic instruments production have led to the widespread contamination of hexavalent chromium (Cr^6+^) in the biosphere, so the bioavailability and biomobility of Cr^6+^ will be increased^11^. Chromium exists mainly in two oxidation states, trivalent and hexavalent; the toxic effects of chromium on the ecosystems and their inhabitants depend on its valence state^12^. Highly poisonous, mutagenic, mobile, and soluble Cr^6+^ ions are typically found in association with oxygen as chromate (CrO_4_^2–^) at pH levels higher than 6.5 or dichromate (Cr_2_O_7_^2–^) at low pH levels^12^. While Cr^3+^ is less toxic, bioelement and usually occurs as Cr(OH)_2_^+^, CrOH^2+^, Cr(OH)_3_, and Cr(OH)_4_^–^, Cr_2_(OH)_2_ and Cr_3_(OH)_4_. However, these industrial activities generated large amounts of solid and liquid waste that was rich in chromium, as well as air emissions^13,14^. The severe and frequent exposure to hexavalent chromium ions can cause many diseases, such as lung and skin cancer^15^, reduction of immune system efficiency, failure in the liver and kidney, internal bleeding, and DNA damage, ulcers in nasal lining nose, irritation, anemia, stomach and small intestine ulcers, and other problems for the respiratory system^16^. Therefore, numerous scientific metal ion elimination strategies have become targeted as possible solutions.

Some physicochemical techniques such as (chemical precipitation, ion exchange, reverse osmosis and electrochemical) are used for chromium removal^4–7,11^. Moreover, bioremediation is the biological approach to degrade heavy metals using indigenous microorganisms such as (bacterial, fungal and yeast and algae) are also used for chromate ions removal^11^. Currently, various techniques for Cr^6+^ uptake are not ecofriendly and also, a large amount and number of chemicals are consumed. Chromium uptake from contaminated sites through adsorption process may be the best technology in present^17,18^. There are various carbonaceous porous materials to remove Cr^6+^ from solution as biochar, activated carbon (AC)^19^, multi-walled carbon nanotubes^20^, activated carbon-supported Fe catalyst and magnetic activated carbon Nano composite were applied^21,22^.

Although the synthesized carbonaceous materials have a large specific surface area, and the high cost of the preparation still prohibits high pore volume, its performance, the lower removal efficiency and the adsorption capacity still need to be improved^23^. To enhance the adsorption performance, a skillful technology was required to functionalize the activated carbon with heteroatoms such as nitrogen, halogen, and oxygen^24,25^. Nitrogen doping activated carbon (NDAC) can be used as a good substitution for the elimination of Cr^6+^. Duan et al.^26^ prepared nitrogen-doped carbon nanosheets from polyurethane foams by hydrothermal carbonization and used them for the adsorption of Cr^6+^ ions with a maximum removal capacity188 mg/g. Sun et al.^27^ developed a new method using nitrogen-doped hierarchical porous carbon resulting from a silkworm cocoon with a maximum removal capacity of 366.3 mg/g. Carboxylated porous carbon with nitrogen-doped was developed as an adsorbent for the uptake of the Cr^6+^ ions from contaminated water (uptake value 104 mg/g)^13^. Wang et al.^28^ prepared an N-doped nanosheet from sewage sludge for adsorption of Cr^6+^ from aqueous solution with *Q*_m_ (7.74 mg/g). Also Abushawish et al.^29^ established a new process for the possible removal of Cr^6+^ ions from water by nitrogen-doped coconut granulated AC with a maximum adsorption capacity (*Q*_m_) 15.15 mg/g. The novelty of this work lies in the utilization of fish waste as a good source of nitrogen to create self-nitrogen-doping activated carbons (NDACs) with high specific surface area, high nitrogen content, and potential application as a better adsorbent for harmful chromium removal from water. The formed NDACs at 600, 700 and 800 °C were characterized with different methods and techniques such as FTIR spectroscopy, TGA, DTA, surface area analysis (BET, BJH, MP, t-plot), SEM, EDX, XRD and XPS analyses. The adsorption of chromium by NDAC was investigated using batch methods and subjected to isotherm and kinetic model studies. The effect of pH, contact time, NDACs different dosages and Cr^6+^ ions different concentrations was also investigated.

## Material and methods

### Formation of NDAC

Self-nitrogen-doped activated carbon (NDAC) was prepared by a hydrothermal process followed by carbonization at high temperatures. Fish waste (mixture of *Atherina hepseetus* and *Sardina pilchardus* of 60% protein) and ZnCl_2_ at a mass ratio (2:1) were mixed in 300 mL distilled water (DW). Then, the uniform mixture was transported into a 500 mL Teflon-lined stainless-steel autoclave and hydrothermally treated at 180 °C for 5 h. Afterwards, the hydrothermal product was put in a mortar and oven dried at 125 °C overnight. Further addition of 50 g of sawdust and 300 mL DW were added to the dried hydrothermal product, and then the uniform mixture was oven dried at 125 °C overnight. The hydrothermal product was put into the high-temperature area of the tube muffle furnace with a flow rate of N_2_ of 100 mL/min. To obtain NDAC with higher surface area, the carbonization temperatures for N-doped activated carbon preparation were 600, 700 and 800 °C^7^. These temperatures were maintained for 1 h under 100 mL/min of nitrogen flow. After the temperature of the tube muffle furnace was let down to cool to 100 °C, the NDAC was collected in 100 mL DW. The black powders were collected by filtering and adequately washing with DW. After that it was refluxed for 2 h in 2N HCl solution. Afterwards, the refluxed NDAC was filtered, washed with DW and then with ethanol and oven dried at 125 °C overnight. Then the last step was sonication which possessed a massive role in the improvement of the obtained NDAC through cleaning pores. 100 mL of DW was added to dried NDAC and utilized in an ultrasonic bath for 0.5 h, decanting solution, washing with 100 mL ethanol, filtration and drying. Finally, the prepared NDACs at 600, 700, and 800 °C were labelled as NDAC600, NDAC700, and NDAC800, respectively^7^.

### Characterizations

For Cr^6+^ ions concentration analysis, a spectrophotometer [Analytic Jena (SPEKOL1300 UV/Visible spectrophotometer)] matched with 1 cm optical glass cell path was used. Shaker [A JS shaker (JSOS-500)], Thermo shaker incubator (GSSI-100 T sh), Tubular Furnace Nabertherm B180 (RT 50/250/13), and JENCO (6173) pH meter were used for the experimental work. Fourier transform infrared spectrometer (FT-IR: Bruker Vertex 70 linked to Platinum ATR model V-100) was used to determine the functional groups and surface chemical state of prepared NDACs. Scanning electron microscope (SEM: LEO, 1450VP), coupled with EDX unit, was applied to inspect the morphology of the NDACs. The volume of monolayer (*V*_m_) (cm^3^ (STP) g^−1^), the surface area (*S*_BET_) (m^2^/g), volume of total pores (*V*_T_) (*p*/*p*_0_) (cm^3^/g), energy constant (*C*), mean diameter of pores (nm) and the average pore radius were calculated according to BET^30^ analysis of the isotherm. The BET surface area (*S*_BET_) analysis of the NDACs was obtained via N_2_ adsorption at 77 K by analyzer instrument (BELSORP—Mini II, BEL Japan, Inc.)^31,32^. Also, the surface area of micropore (*S*_mi_) and volume of micropore (*V*_mi_) as well as the surface area of mesopore (*S*_mes_) and volume of mesopore (*V*_mes_) of NDACs were determined by the Barrett-Joyner–Halenda (BJH) methods, following the BELSORP analysis program software. The distribution of pore size was measured from desorption isotherm via applying the BJH method^30^. Thermal analyses were used to define the thermal stability of sawdust, fish waste and fish-waste/sawdust/ZnCl_2_ hydrothermal mixture using the SDT650-Simultaneous Thermal Analyzer device at a temperature range of 25 to 1000 °C at a temperature ramp rate of 10 °C/min under 100 mL/min of nitrogen gas flow^9^. D2 PHASER Instrument, manufactured by Bruker in Germany, was used for the XRD analysis^33^. Elemental analysis was performed using a Thermo Fisher Scientific K-Alpha XPS with a pass energy of 50 eV at a base pressure of ~ 10^–9^ mbar.

### Batch adsorption experiment for hexavalent chromium

A stock solution containing 1000 mg/L of Cr^6+^ was prepared by dissolving 2.8289 g of K_2_Cr_2_O_7_ in 100 mL of DW and completed to 1 L using DW, the initial concentrations of Cr^6+^ ions solutions (100—400 mg/L) was prepared by dilution of this stock solution. The adsorption study of Cr^6+^ was carried out using a batch adsorption process^34^. 100 mL of the Cr^6+^ ions solution was agitated with various doses of the prepared NDAC in a shaker (JSOS- 500). The solution was examined for the remaining Cr^6+^ ions concentration using a visible–UV spectrophotometer at the wavelength (λmax 545 nm) and 1,5–diphenylcarbazide as a reagent. The adsorption capacities of NDAC can be measured using Eq. (1).

1 $${q}_{t}=\frac{\left({C}_{0}-{C}_{t}\right)}{W}\times V,$$

where *q*_t_ is the adsorption capacity (mg/g) of the adsorbent at time *t*; *C*_0_ is the initial concentration (mg/L) of pollutant; *C*_t_ is the remaining concentration of the pollutant after adsorption had taken place over a period of time *t* (mg/L); *V* (L) is the volume in liter of the pollutant solution and *W* (g) is the mass of adsorbent in gram. The removal % of Cr^6+^ ions from water is measured from Eq. (2).

2 $$Removal \,\%=\frac{\left({C}_{0}-{C}_{t}\right)}{{C}_{0}}\times 100.$$

The pH influence on Cr^6+^ ions adsorption was investigated by mixing 100 mg of the NDAC600 to 100 mL of 100 mg/L of Cr^6+^ ions solution with initial pH values 1.5, 3, 5, 7, 9 and 11. The pH values of the solution were attuned with a solution of 0.1 M HCl and 0.1 M NaOH. The mixtures were shaken at 200 rpm for 2 h at room temperature and sampled for Cr^6+^ ions concentration analysis. The isotherm study and the impact of adsorbent doses on Cr^6+^ ions adsorption were achieved using various initial concentrations of Cr^6+^ ions water solutions (100, 150, 200, 250 and 400 mg/L) using different weights of NDAC600 (0.5, 1.0, 1.5, 2.0 and 2.5 g/L Cr^6+^ solution) were shaken at 200 rpm for 10, 15, 30, 45, 60, 90 and 120 min at 25 ± 2 °C^34^.

### Point of zero charges (pH_PZC_)

**Figure 1:**
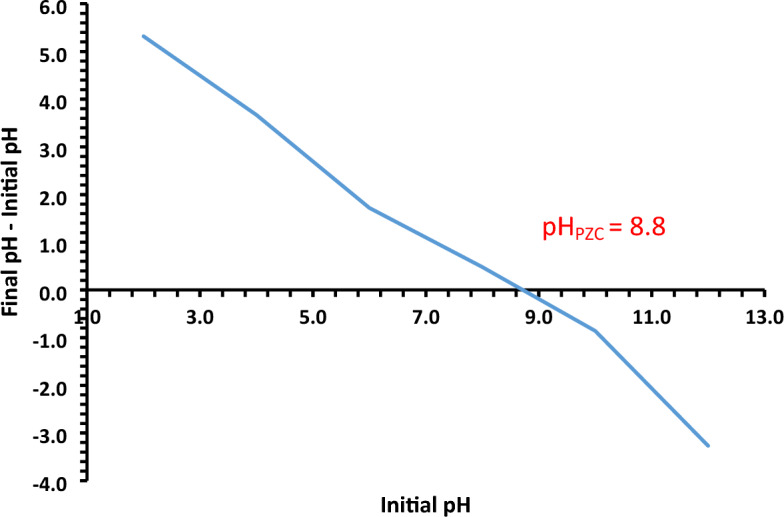
The pH_PZC_ of the NDAC600 at 25 ± 2 °C.

To study the attraction and repulsion forces between adsorbent and adsorbate during the removal process, the pH of pH_PZC_ should be studied. The approach outlined in the literature was used to obtain the pH_PZC_^3,35,36^. In brief, in 100 mL flasks, 50 mg of NDAC600 was taken in 50 mL of 0.1 M NaNO_3_ solutions. The initial pH solution (pH_i_) was adjusted to a value ranged from 2 to 12 using 0.1 M HCl or NaOH and shaken 24 h. Then the final pH of the supernatant liquid (pH_F_) was calculated. Moreover, the variance at the initial and final pHs (*Δ*pH = pH_i_ − pH_F_) was plotted against the pH_i_, Eq. (3). The pH value at the ΔpH equaled zero was ascribed as pH_PZC_ of the adsorbent. The pHzpc value of NDAC600 was reported to be 8.8 (Fig. 1). The result designates that, below this pH value, the surface of the NDAC600 has a positive charge due to the protonation of nitrogen atoms into NDACH^+^.

3 $$\Delta \mathrm{pH}={\mathrm{pH}}_{\mathrm{i}}-{\mathrm{pH}}_{\mathrm{F}}$$

### Adsorption isotherms

The linearized Langmuir (LIM) (Eq. (4)), Freundlich (FIM) (Eq. (5)), Temkin (TIM) (Eq. (6)), Dubinin–Raduskevich (DRIM) (Eq. (7)) and Halsey isotherm (HIM) (Eq. (8)) models were implemented for the sorption isotherms of NDAC600 for hexavalent chromium (Cr^6+^) ions to estimate the distribution of Cr^6+^ ions in both solid and liquid phases when the adsorption process reached the equilibrium^37^. The adsorption isotherm investigations were carried out using various initial concentrations of Cr^6+^ ions (100–400 mg/L) at 25 ± 2 °C onto NDAC600 adsorbent doses (0.5–2.5 g/L).

4 $$\frac{{C}_{e}}{{q}_{e}}=\frac{1}{{Q}_{m}{K}_{L}}+\frac{1}{{Q}_{m}}\times {C}_{e},$$

where *Q*_m_ (mg/g) is the mono-layer maximum adsorption capacity of NDAC600, *K*_L_ (L/mg) is the LIM adsorption constant, and *q*_e_ (mg/g) is the adsorption capacity of NDAC600 at equilibrium. Consequently, a plot of *C*_e_/*q*_e_ against *C*_e_ gives a straight line of intercepts 1/(*Q*_m_*K*_L_) and slope 1/*Q*_m_.

5 $$\mathrm{log}{q}_{e}=\mathrm{log}{K}_{F}+\frac{1}{n}\mathrm{log}{C}_{e}$$

where *K*_F_ and *n* are the FIM adsorption constants, which can be obtained from the linear plot of log *q*_e_ against log *C*_e_.

6 $${q}_{e}=\frac{RT}{B}\mathrm{ln}A+\frac{RT}{B}\mathrm{ln}{C}_{e},$$

where *A* and *B* are TIM constants, *R* is the gas constant, and *T* is the absolute temperature. *A* plot of *q*_e_ against ln *C*_e_ can be used to calculate *A* and *B* constants.

7 $$\mathrm{ln}{q}_{e}=\mathrm{ln}{q}_{m}-\beta {\varepsilon }^{2},$$

where *β* is a coefficient related to the adsorption mean free energy (mmol^2^/J^2^), *q*_m_ is the maximum adsorption capacity, and *ε* is the polanyi potential (J/mmol) that can be written as: *ε* = RT (1 + 1/*C*_e_).

8 $$\mathrm{ln}{q}_{e}=\frac{1}{n}\mathrm{ln}k+\frac{1}{n}\mathrm{ln}{C}_{e},$$

where *K* and *n* are the HIM constants, which can be calculated from the linear plot of ln *q*_e_ against ln *C*_e_.

### Error functions

To define the adsorption model that best designates the interaction between the NDAC600 and Cr^6+^ ions, the fit goodness is used. The sum-of-squared errors (ERRSQ) (Eq. 9), average relative error (ARE) (Eq. 10), non-linear chi-square test (X^2^) (Eq. 11), hybrid fractional error function (HYBRID) (Eq. 12), Marquardt’s percent standard deviation (MPSD) (Eq. 13), Average percentage error (APE%) (Eq. 14), Root Mean Square (RMS) (Eq. 15) and the sum of absolute errors (EABS) (Eq. 16) are some of the error functions that have been used to study model fit analysis of the isotherm models of the removal of Cr^6+^ ions of various starting concentration (100–400 mg/L) at 25 ± 2 °C onto NDAC600 doses (0.5–2.5 g/L) (Table 1)^37–41^.

**Table 1:** EF models used to examine the isotherm models applicability.

Error model name	Error equation	Equation no
ERRSQ	$ERRSQ= \sum_{i=1}^{n}{\left({q}_{e,isotherm}-{q}_{e,cal}\right)}^{2}$	(9)
ARE	$ARE= \frac{100}{n}+\sum_{i=1}^{n}\left|\frac{{q}_{e,isotherm}-{q}_{e,cal}}{{q}_{e,isotherm}}\right|i$	(10)
X^2^	${X}^{2}= \sum_{i=1}^{n}\left(\frac{\left(qemeas-qecal\right)^2}{qeexp}\right)$	(11)
HYBRID	$Hybrid= \frac{100}{p-n} \sum_{i=1}^{p}\left(\frac{{\left({q}_{e, isotherm}-{q}_{e, cal}\right)}^{2}}{{q}_{e,isotherm}}\right)i$	(12)
MPSD	$MPSD=100\times \sqrt{\frac{\sum_{i=1}^{n}{\left(\frac{{q}_{e, isotherm}-{q}_{e, cal}}{{q}_{e,isotherm}}\right)}^{2}}{n-p}}$	(13)
APE	$APE\%= \frac{100}{n}\times \sum_{i=1}^{n}\left|\frac{{q}_{e,isotherm}-{q}_{e,cal}}{{q}_{e,isotherm}}\right|i$	(14)
RMS	$RMS=100\times \sqrt{\frac{\sum_{i=1}^{n}{\left(1-\frac{{q}_{e, cal}}{{q}_{e, isotherm}}\right)}^{2}}{n}}$	(15)
EABS	$EABS= \sum_{i=1}^{n}\left|{q}_{e,isotherm}-{q}_{e,cal}\right|i$	(16)

### Adsorption kinetics

The kinetic models such as the pseudo-first-order (PFOM) (Eq. 17), pseudo-second-order (PSOM) (Eq. 18), Elovich (EM) (Eq. 19), intraparticle diffusion (IPDM) (Eq. 20) and film diffusion (FDM) (Eq. 21) kinetic models were employed to investigate the mechanism and the removal process of toxic chromium ions by NDAC600 (Table 2). The kinetic experiments of Cr^6+^ ions adsorption onto NDAC600 were performed by using various adsorbent doses (0.50–2.50 g/L) and various starting Cr^6+^ ions concentrations (100–400 mg/L)^37,42–44^.

**Table 2:** The used adsorption kinetic models equations.

Model name	Model equation	Equation no
PFOM	$\mathrm{log}\left({q}_{e}-{q}_{t}\right)=\mathrm{log}{q}_{e}-\frac{{K}_{1}}{2.303}t$	(17)
PSOM	$\frac{t}{{q}_{t}}=\frac{1}{{K}_{2}{q}_{e}^{2}}+\frac{t}{{q}_{e}}$	(18)
EM	${q}_{t}=\frac{1}{\beta }\mathit{ln}\left(\propto \beta \right)+\frac{1}{\beta }ln(t)$	(19)
IPDM	${q}_{t}={K}_{dif}{t}^{0.5}+C$	(20)
FDM	$\mathit{ln}\left(1-F\right)={K}_{FD}(t)$	(21)

The *q*_t_ and *q*_e_ (mg/g) are the quantities of ions adsorbed at time *t* and at equilibrium, respectively, and *k*_1_ (min^−1^) is the PFO rate constant of the adsorption process. The *k*_2_ (g/mg min) is the PSOM equilibrium rate constant of adsorption. The *α* (mg/g min) is the initial sorption rate constant, and the parameter *β* (g/mg) is related to the extent of surface coverage and activation energy for chemisorption. The *k*_dif_ (mg/g min^0.5^) is the IPDM rate constant. The *K*_FD_ is the external film mass transfer coefficient.

## Results and discussion

### Characterization of self-Nitrogen-doping activated carbon

#### SEM analysis

**Figure 2:**
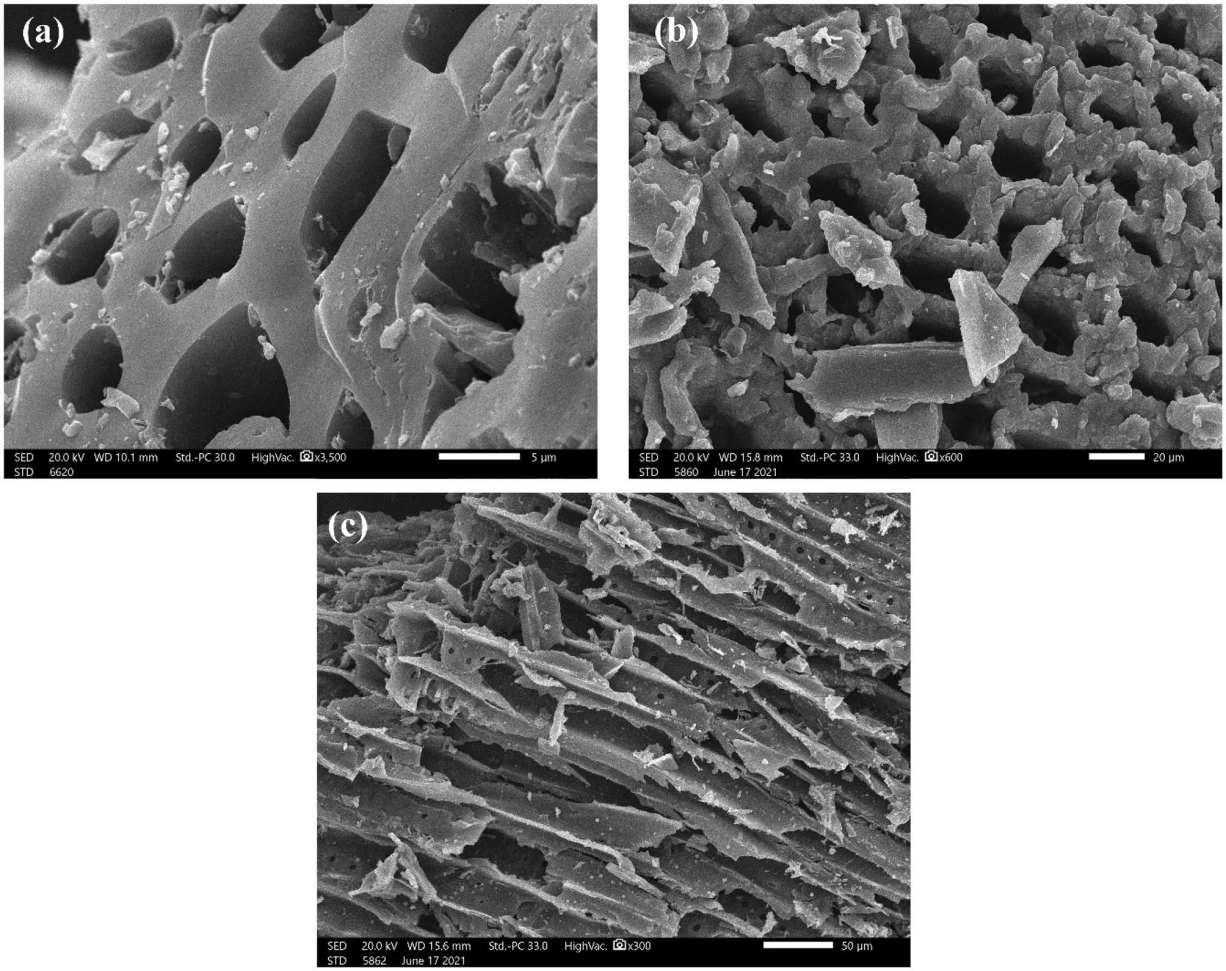
The SEM image of (**a**) NDAC600, (**b**) NDAC700, (**c**) NDAC800.

The morphology of NDAC600, NDAC700 and NDAC800 was proved by using SEM analysis. As reported in Fig. 2a, NDAC600 image has a smooth surface and many micropores, indicating a unique homogenous porous structure. It can be observed that from Fig. 2b, the NDAC700 image possessed a considerable amount of clear nano-channels structure with micropores. As the pyrolysis temperature increased to 800 °C, the nano-channels structure of NDAC800 was broken, and the surface fluctuated (Fig. 2c)^34^.

#### Pore structure analysis

**Figure 3:**
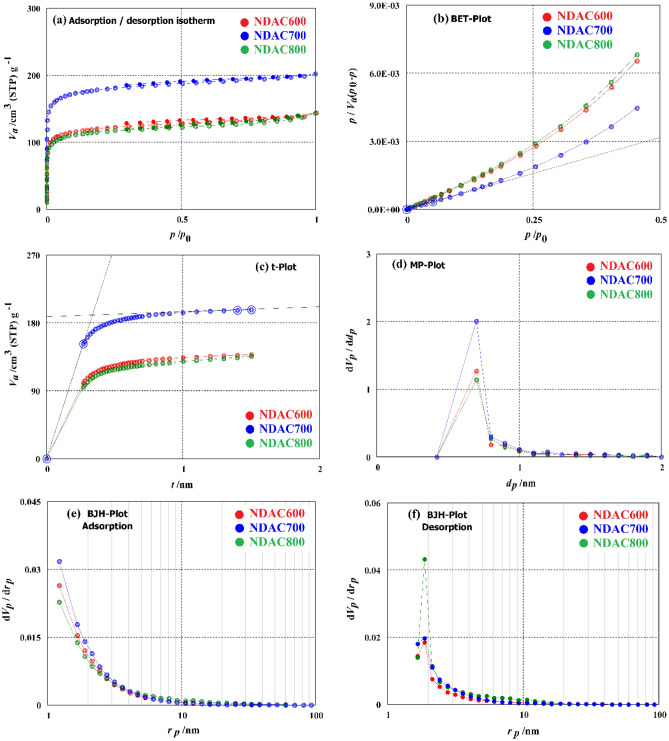
Surface area analysis (**a**) Adsorption–desorption curve, (**b**) BET analysis curve, (**c**) *t*-plot analysis curve, (**d**) MP analysis curve, (**e**) BJH adsorption analysis curve, (**f**) BJH desorption analysis curve.

The microporous structure of NDACs was further established by the N_2_ adsorption–desorption analysis. The nitrogen adsorption–desorption isotherms of NDAC600, NDAC700 and NDAC800 were plotted in Fig. 3a. As shown in Fig. 3a, the trend of the curve of volume adsorbed for all the NDACs was very similar, and the shape of isotherm attributed to type I, denoting that the NDACs were essentially microporous. The analyses of the N_2_ isotherms were performed by applying the BET, *t*-plot, MP, BJH adsorption and BJH desorption process. The BET surface area, total pore volume and mean pore diameter of the NDAC at 600, 700 and 800 °C measured by N_2_ adsorption − desorption isotherms illustrated in (Fig. 3b and Table 3). The BET surface area of the NDAC at 600, 700 and 800 °C was 455.22, 680.86 and 437.51 m^2^/g, respectively. Additionally, the total volume of pores of the NDAC at 600, 700 and 800 °C was 0.2206, 0.3116 and 0.2202 cm^3^/g while the mean pore diameter was 1.9386, 1.8305 and 2.0133 nm, respectively. As shown in Table 3, the NDAC at 700 °C has a higher surface area and total volume of pores, but it has a lower mean pore volume value than the NDAC at 600 and 800 °C. This result may have been attributed to the collapse of the pore structure during the carbonization step when the temperature rose from 700 to 800 °C. The *t*-plot technique has been used to determine the microporous surfaces and if the *t*-plot graph is a straight line, then the sample has no pores. The *t*-plot curve of the NDAC at 600, 700 and 800 °C are plotted in Fig. 3c. As shown in Fig. 3c, the *t*-plot curve of the NDAC at 600, 700 and 800 °C is not a straight line; the first segment of the graph is illustrated the adsorption due to the micropore filling while the second segment occurs due to the adsorption of the external surface. From *t*-plot graph, the average thickness of the adsorbed layer on the surface (*t*), total surface area (*a*_1_), external surface area (*a*_2_), average pore diameter (2*t*) and pore surface area (*a*_1_–*a*_2_) can be measured (Table 1). The average pore diameter of NDAC at 600, 700 and 800 °C were 0.6941, 0.6754 and 0.6726 nm, respectively. From the results we can deduced that, the average pore diameter (2*t*) value is smaller than 0.7 nm, so the pore is micropore. The MP-plot is an analysis method obtained from a *t*-plot procedure and applied for defining the presence or nonexistence of micropores and their size distribution. Figure 3d shows the pore size distribution of NDAC at 600, 700 and 800 °C as obtained from Mp-plot analysis. The total specific surface area (*a*_1_), the external specific surface area (*a*_2_), the specific surface area (*a*_1_–*a*_2_), and the pore volume (*V*_p_) data can be found in Table 3. It can be seen from Fig. 3d, NDAC at 600, 700 and 800 °C have micropores of 0.4 to 0.9 nm diameter (*d*_p_), and have a distribution peak (*d*_p,peak_ nm) at 0.7 nm. The specific surface area of NDAC at 600, 700 and 800 °C was 508.65, 771.59 and 480.033 m^2^/g while the volume of pores (*V*_p_) was 0.2023, 0.3011 and 0.1887 cm^3^/g. However, all the NDAC at 600, 700 and 800 °C samples showed a small pore size and narrow size distribution curve where it has a high specific surface area. This result harmonizes well with the SEM analysis. The Fig. 3e, f and Table 3, show the result of BJH adsorption and desorption analyses of NDAC at 600, 700 and 800 °C. The results indicate that the NDAC at 600, 700 and 800 °C samples have very little specific surface area of mesopores.

**Table 3:** Surface area analysis of prepared self-Nitrogen doping activated carbons (NDAC600, NDAC700, and NDAC800.

Analysis method	Sample entry	NDAC600	NDAC700	NDAC800
	AC yield (%)	24.10	27.00	31.88
BET	*S*_BET_ (m^2^/g)	455.22	680.86	437.51
	*V*_m_ (cm^3^/g)	104.59	156.43	100.52
	*D*_p_ (nm)	1.9386	1.8305	2.0133
	*V*_T_ (cm^3^/g)	0.2206	0.3116	0.2202
*t*-plot	*S*_mi_ (m^2^/g) (*a*_1_–*a*_2_)	565.193	859.2313	517.032
	*V*_mi_ (cm^3^/g)	0.1973	0.2919	0.1749
	2*t* (nm)	0.6941	0.6754	0.6726
MP	*a*_1_–*a*_2_ (m^2^ ∕g)	508.646	771.59	480.033
	*V*_p_ (cm^3^∕g)	0.2023	0.3011	0.1887
BJH ads	*V*_me_ (cm^3^/g)	0.0532	0.0573	0.0591
	*S*_me_ (m^2^/g)	48.020	56.104	44.516
BJH des	*V*_*me*_ (cm^3^/g)	0.0277	0.03142	0.04649
	*S*_me_ (m^2^/g)	16.33	20.330	27.728

#### Thermogravimetric and differential thermal analyses (TGA and DTA)

**Figure 4:**
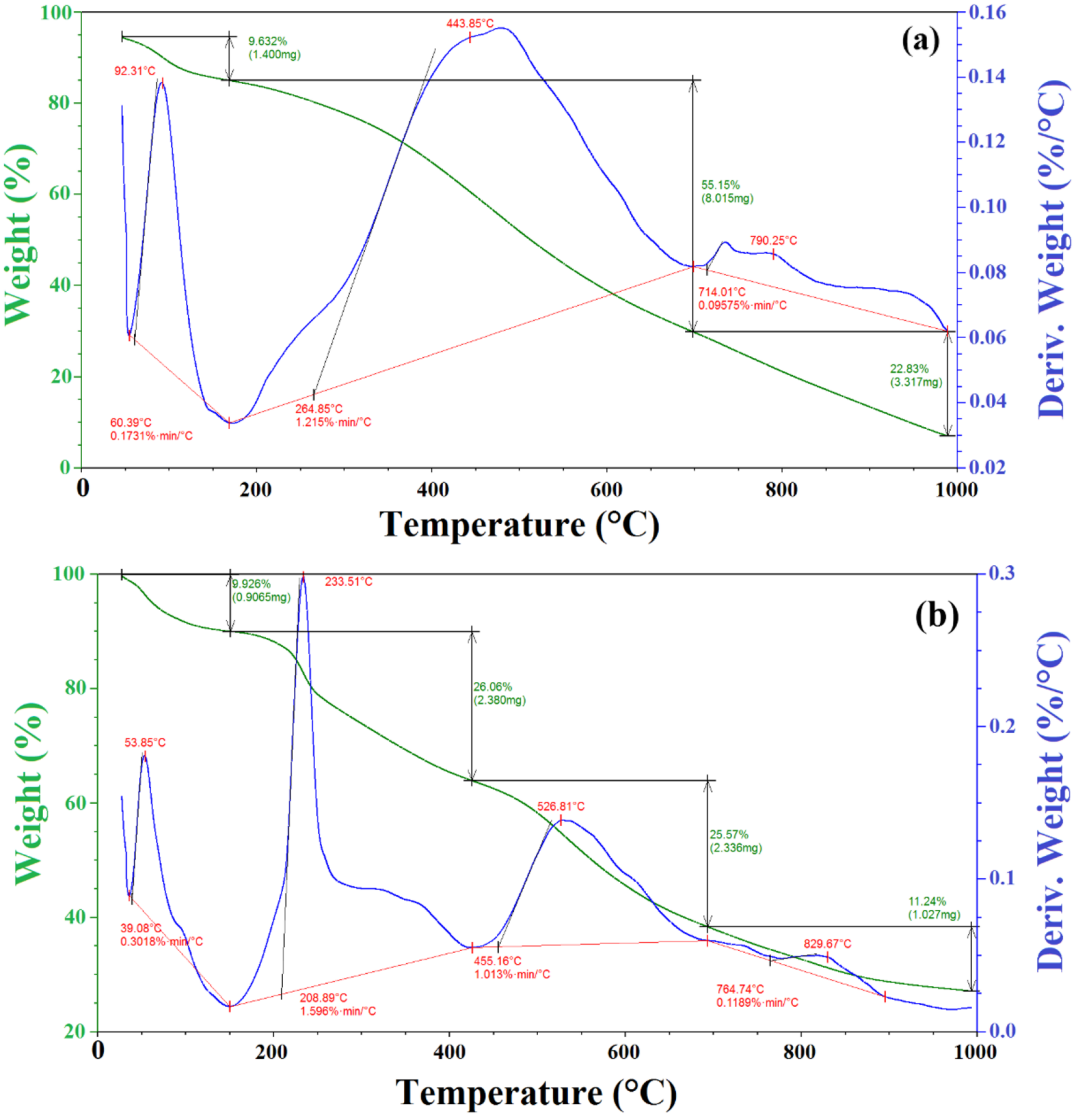
TGA and DTA analysis (**a**) Sawdust analysis, (**b**) Fish waste/Sawdust/ZnCl_2_ analysis.

The TGA investigation was used to estimate solid matter's thermal stability and decomposition process. The thermal loss curve and differential thermal analysis of the sawdust are shown in Fig. 4a. A slightly weight loss (9.632%) appeared between 60.39 and 160 °C due to bound moisture. At temperatures around 160 °C and 714 °C, the sawdust shows a significant weight loss (55.15%) owing to the degradation of hemicellulose and cellulose^35^. The main degradation process with a maximum peak as shown in the Fig. 4a takes place at 443.85 °C. The last weight loss of the sawdust was relatively gentle above 714 °C, and its value was 22.83% between 714.01 and 980 °C. The maximum weight loss occurs at around 790.25 °C and a residue of 12.388% is obtained. The TGA and DTG curves of the fish waste, ZnCl_2_ and sawdust are presented in Fig. 4b. The fish waste, ZnCl_2_ and sawdust exhibited five decomposition stages. In initial stage, the weight loss (5.384%) occurs between 39.62 and 160 °C due to the bound water in sawdust, fish waste and ZnCl_2_ as a hygroscopic substance. In the second stage, around 222.50 and 295.50 °C, the weight loss 18.03% of the total volatiles evolved from fundamental lipid decomposition compounds in fish waste, such as aldehydes (CHO) and ketones (C = O). In the third stage, from 295.50 to 501.15 °C, a weight loss of 15.65% of the volatiles was released, representing the degradation of the fatty acid hydrocarbon chains and the protein component of the fish waste constituent sample. Additionally, in the third stage, below 400 °C, ZnCl_2_ melts at 320 while above 400 °C, the molten ZnCl_2_ will be vaporized. Also, across this temperature range particularly hemicellulose and cellulose will be degraded as a major component of sawdust. However, lignin has higher thermal stability than hemicellulose and cellulose, and the range of its degradation is extensive^45^. In the fourth and fifth stages, within the temperatures ranging from 501.15 °C to 834.09 °C and around 834.09 to 990.0 °C, respectively, the meal fish, ZnCl_2_ and sawdust sample exhibited weight loss 23.95 and 9.413%, respectively, of its volatiles and a residue of 27.573% will be formed^46^.

#### Analysis using energy-dispersive X-ray spectroscopy (EDX)

**Figure 5:**
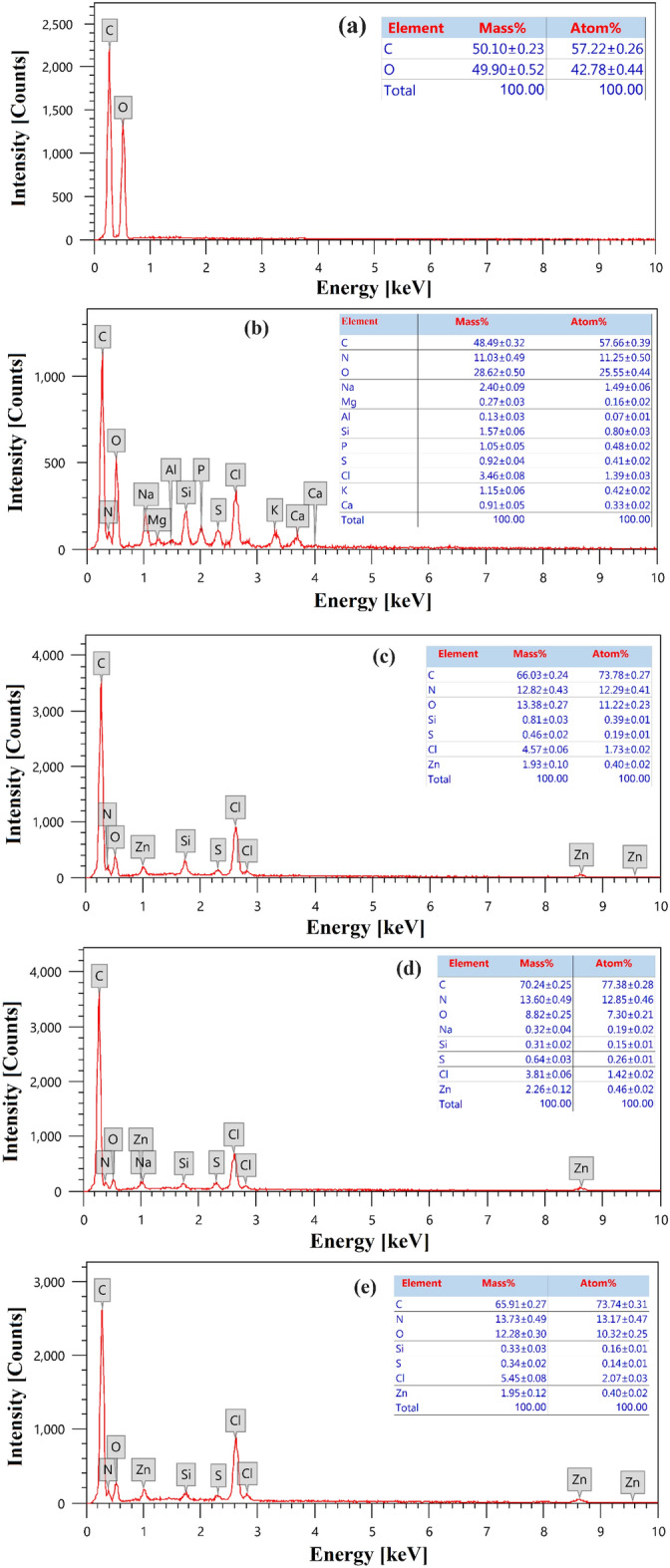
EDX element analysis of (**a**) Sawdust analysis, (**b**) Fish waste analysis, (**c**) NDAC600 analysis, (**d**) NDAC700 analysis, (**e**) NDAC800 analysis.

The surface contents of carbon, hydrogen and nitrogen of the self-doping activated carbons NDAC600, NDAC700, and NDAC800 are shown in Fig. 5a–c, respectively. Additionally, the elemental analyses of sawdust and fish waste (60%, protein) are shown in Fig. 5d,e. As can be seen from Fig. 5a, the C, N, and O weight percentages of NDAC600 were 66.03, 12.82 and 13.38%, respectively, which appear to be compatible with the atomic percentages of 73.78% (C), 12.29% (N), and 11.22% (O). The weight percentages of C, N, and O of NDAC700 were 70.24, 13.60 and 8.82% with atomic percentages of 77.38, 12.85 and 7.30%, and for NDAC800 were, 65.91, 13.73 and 12.28% with the atomic percentages of 73.74, 13.17 and 10.32%, respectively. By comparing with Fig. 5d,e, the sawdust had no nitrogen content, while the fish waste has 11.03% as nitrogen dopant with atomic percentages of 11.25%; it means that after hydrothermal and pyrolysis processes, the nitrogen element was introduced onto the surface of adsorbents of self-nitrogen doping activated carbons NDAC600, NDAC 700, and NDAC800.

#### Analysis using X-Ray diffraction (XRD) analysis

**Figure 6:**
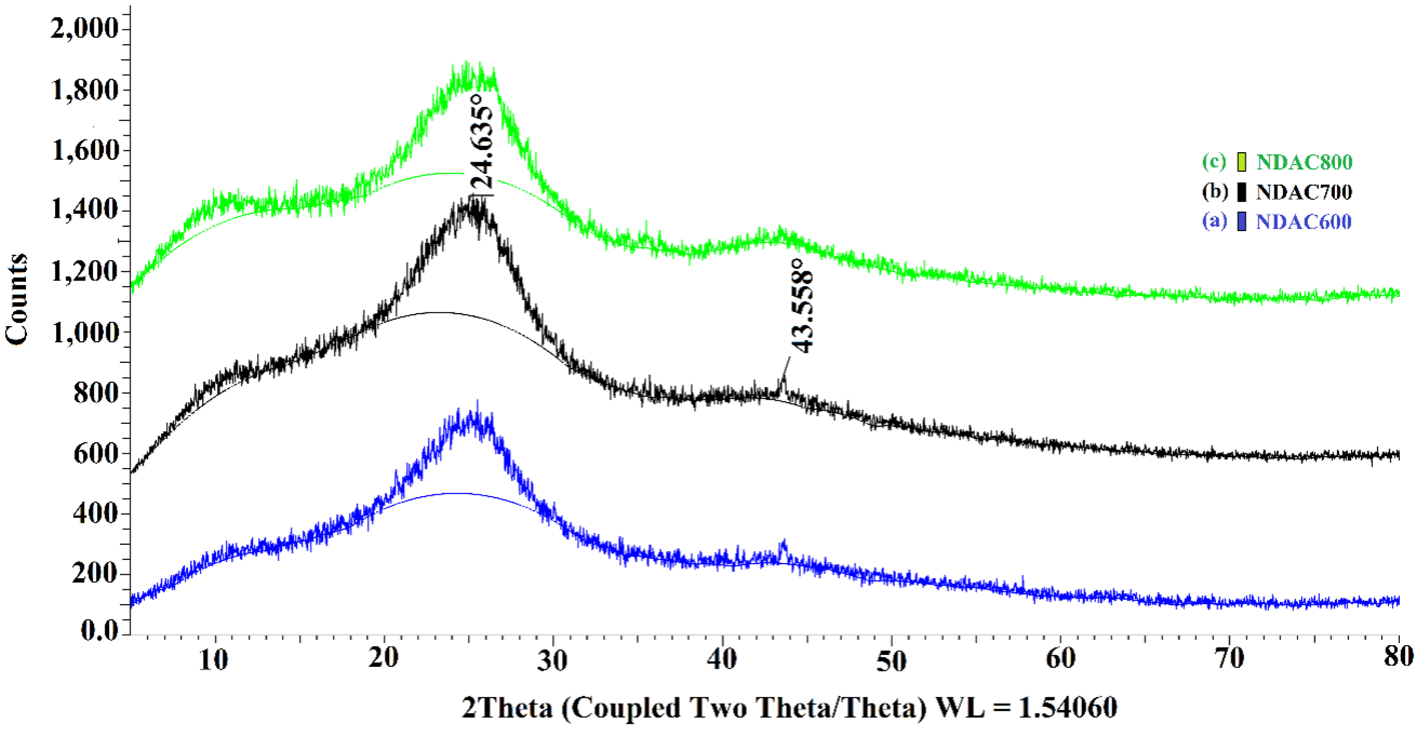
XRD analysis of (**a**) NDAC600, (**b**) NDAC700, (**c**) NDAC800.

XRD analysis of the fabricated NDAC600, NDAC700 and NDAC800 are presented in Fig. 6. The XRD spectra of all nitrogen-doped activated carbons reflect two peaks around 24.635° and 43.558° assigned to the (002) and (101) plans of carbons, respectively. In this case, the weak intensity peak would reveal the smaller crystallites, which is convenient with the amorphous structure of NDACs^35,36^.

#### X-ray photoelectron spectroscopy (XPS)

**Figure 7:**
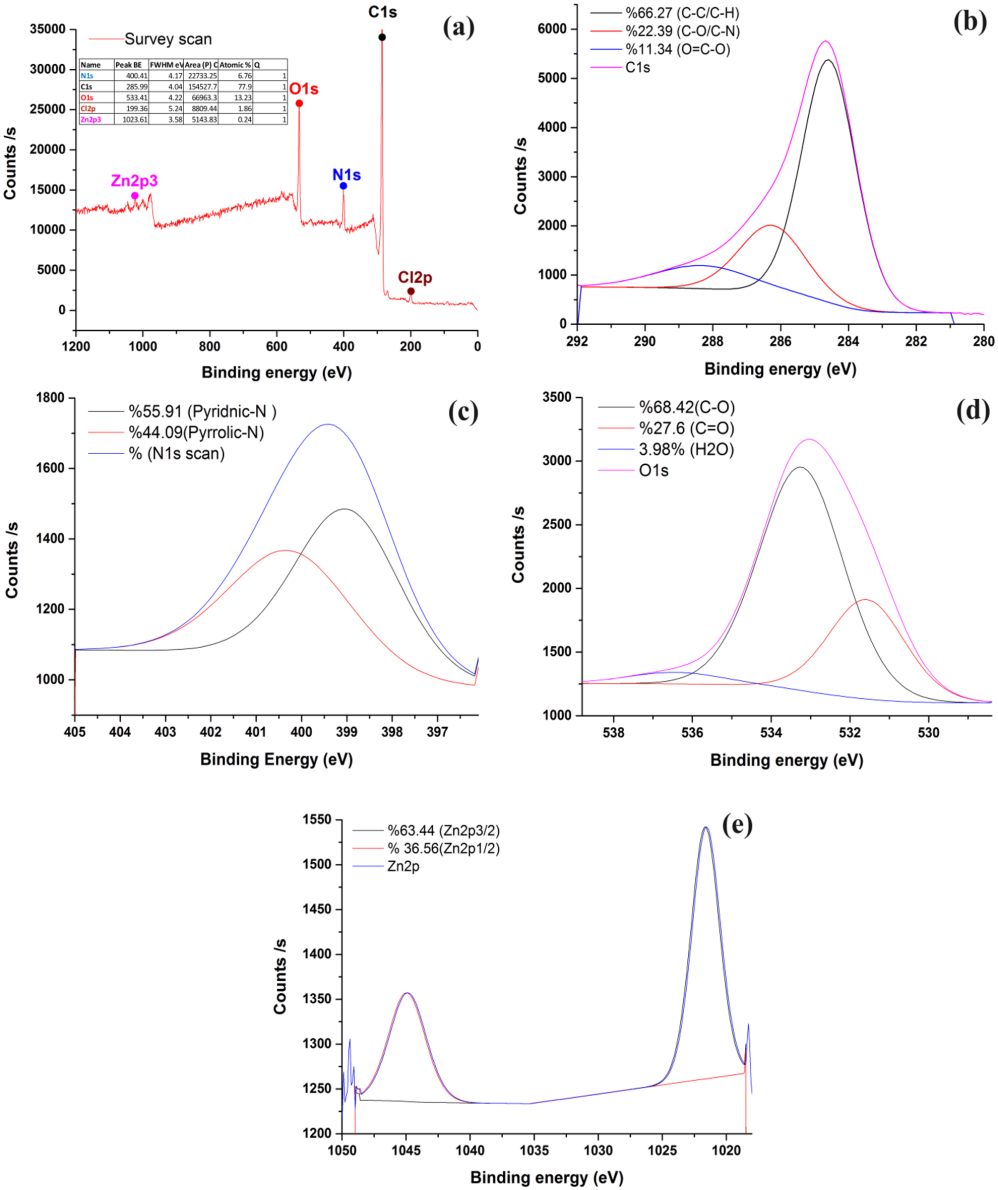
High resolution of XPS core level spectra of (**a**) full spectrum, (**b**) C1s, (**c**) N1s, (**d**) O1s and (**e**) Zn2p.

XPS was used to qualitatively analyze the functional groups on the surface of activated carbon^47,48^. Figure 7a is the wide full XPS spectra of precursor and NDAC600. As seen from the graphs, characteristic peaks of C1s, O1s, N1s and Zn2p are found in NDAC600, witnessing that N have been successfully retain on NDAC. The peaks located in 285.99, 400.41, 533.41 and 1023.61 eV are corresponding with C1s, N1s, O1s and Zn2p, respectively. Figure 7b C1s exhibits three peaks by curve fitting of the C1s spectrum. The C1s spectrum can be deconvoluted into three peaks centered at 284.58 (66.27%), 286.26 (22.39%), and 288.31 eV (11.34%), assigned to sp^2^-C hybridized C = C bonds, C − O/C − N bonds and − O/C = O bonds, respectively^48–51^. The N1s XPS spectra of NDAC600 could be deconvoluted into two types of N-containing compounds, and results are depicted in Fig. 7c. The peaks of N1s located in 398.98 (pyridinic N) and 400.22 (pyrrolic N)^51,52^, respectively in NDAC600. The presence of pyridinic and pyrrolic N promotes the ion transport from the electrolyte to electrode material, effectively enhancing the capacitive properties. The O1s XPS spectrum of the NDAC600 exhibits three peaks shown in Fig. 7d at 531.08 eV (27.6%), 532.74 eV (68.42) and 535.93 eV (3.98), corresponding to (C = O), (C-O) and (H_2_O). The Zn2p peak of NDAC600 is shown in Fig. 7e, and the binding energies of 1044.96 and 1021.66 eV corresponded to the peaks of Zn 2p_1/2_ and Zn 2p_3/2_ of ZnO^53^.

#### The FTIR analysis

**Figure 8:**
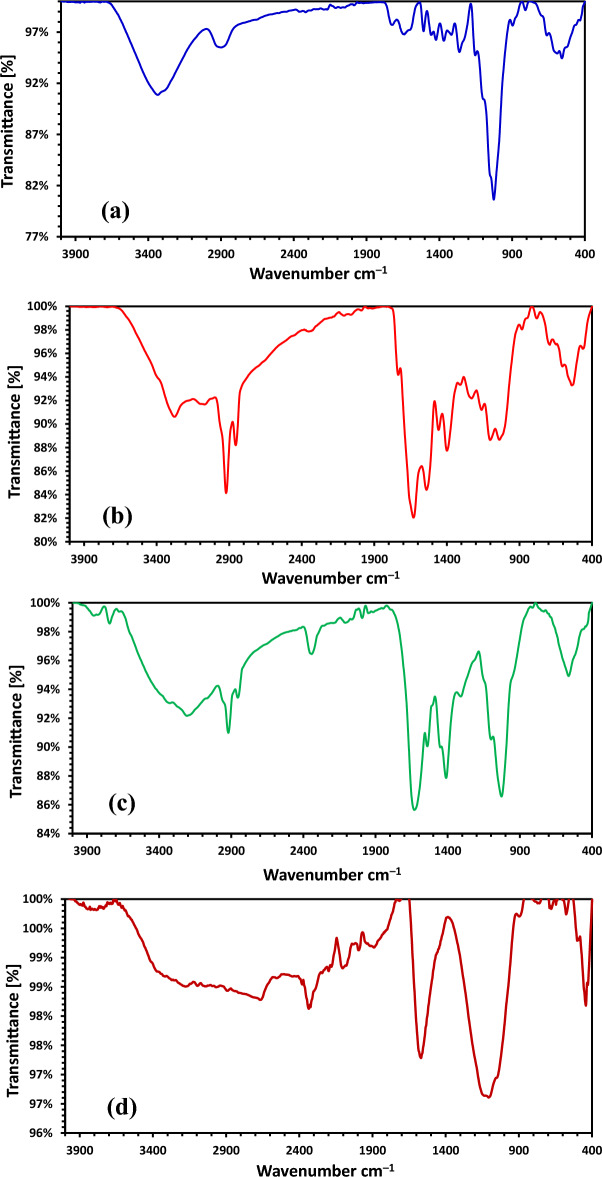
FTIR investigation of (**a**) Sawdust, (**b**) Fish waste, (**c**) Fish waste/Sawdust/ZnCl_2_ hydrothermal, (**d**) NDAC600.

The FTIR spectrum of sawdust is shown a number of absorption bands revealed to the complex composition of the sawdust biomass, which consists of cellulose, hemicelluloses, lignin, pectin and extractives such as fat, waxes, etc. (Fig. 8a). The presence of broad adsorption peak at 3334.65 cm^−1^ was assigned to –OH group of phenolic (lignin and extractives) and hydroxylic (cellulose, hemicelluloses, lignin, extractives and pectin). The weak band at 2899.43 cm^−1^ was ascribed to the stretching vibration of C–H of –CH_2_ group. The appearance of weak bands at 1723.56 and 1639.45 cm^−1^ recognized to C = O stretching (lignin, pectin) and to N–H, respectively. The weak bands at 1456.53 and 1422.96 cm^−1^ were allocated to the symmetric bending of CH_3_. The strong sharp band observed at 1026.09 cm^−1^ can be assigned to C–O stretching of COOH (hemicelluloses, pectin and lignin). The FTIR spectrum of fish meal which was a mixture of *Atherina hepseetus* and *Sardina Pilchardus* (60% protein) was shown in Fig. 8b. An amide-A band was found at a wavenumber of 3276.49 cm^–1^, which designates the occurrence of hydrogen bond between the N–H group with a C = O of the peptide chain. Amide-B adsorption band was observed at a wavenumber of 2921.10 cm^–1^ can be allocated to asymmetrical stretching of CH_2_. The amide-I adsorption band that occurred at 1630.19 cm^–1^ can be assigned to the peptide secondary structure and hydrogen bonding between N–H stretch and carbonyl group in gelatin. The amide-II adsorption band of fish meal was found at 1540.22 cm^–1^ while the amide-III band was detected at 1229.75 cm^–1^; these two significant bands can be represented to N–H bending vibration coupled with C–N stretching vibration and C–H stretching. To determine the functional groups involved in the hydrothermal product of mixture of fish meal, ZnCl_2_, sawdust and water with a mass ratio (2:1:1:12), a comparison between the FTIR spectra before and after the hydrothermal process was done (Fig. 8c). As can be seen from Fig. 8c, the occurrence of a band at wavenumber of 3208.56 cm^–1^ attributed to –OH group in sawdust and amide-A which becoming broader. The moderate band at 2920.32 cm^−1^ was ascribed to the stretching vibration of C–H and amide-B. The disappearance of the peak at wavenumber 1723.59 cm^–1^, which matching to –C = O stretching vibrations from lignin aromatic groups. The peak at wavenumber 1631.66 cm^–1^ was observed as sharper, which reflects N–H or C = C in sawdust and amide-I.

This diminished obviously of the peak at wavenumber 1723.59 cm^–1^ and the appearance of the peak sharper at wavenumber 1631.66 cm^–1^ may be explained by the fact that the condensation reaction has occurred between C = O of lignin aromatic groups and N–H amide group of protein in an available acidic condition in the presence of ZnCl_2_ to form the C = N group. The weak band at wavenumber 1541.32 cm^−1^ was ascribed to amide-II. While there was a sharp peak was found at wavenumber 1410.95 cm^–1^, which was assigned to symmetric bending of CH_3_ in sawdust. Additionally, the sharp band at wavenumber 1027.22 cm^−1^ can be attributed to the C–O stretching of COOH in sawdust. Finally, the disappearance of a peak at wavenumber 1229.75 cm^–1^ represents amide-III. The FTIR spectrum of NDAC600 is shown in Fig. 8d. The broadband with a low intensity that extended from 3175.63 to 3096.27 cm^–1^ region may be assigned with overlapping bands of O–H and N–H stretching vibrations as surface groups that lead to hydrogen bonds formation. The appearance of C–H stretching broad low-intensity peak at 2890.45 cm^–1^. The bands at 2379.60, 2335.18 and 2323.20 cm^–1^ are assigned to C≡N stretching vibrations of isonitrile cyano terminal groups. Two medium peaks relevant to allene (C = C = C) and ketamine (C = C = N) groups have appeared at 2199.62 and 2104.21 cm^–1^. A sharp band at 1568.17 cm^–1^ was observed, ascribed to the N–H group for NDAC600 sample. A comparison of spectra for sawdust, fish waste, the hydrothermal mixture of fishmeal, ZnCl_2_, and sawdust in water with NDAC600 shows obvious changes in the intensity of the band relevant to N–H group. Also, the observed strong band at wavenumber 1106.11 cm^−1^ with a higher shifting from 1027.22 to 1106.11 cm^–1^ can be ascribed to sp^2^ structure of the carbon atoms and recognized to the C–N stretching vibrations proved the successful formation of nitrogen doping activated carbon^54–56^.

### Adsorption studies of Cr^6+^

#### Influence of pyrolysis temperature

**Figure 9:**
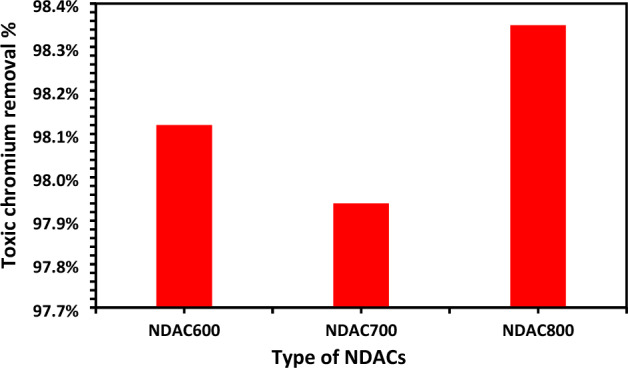
Removal efficiency % investigation of NDAC600, NDAC700, and NDAC800.

The impact of pyrolysis temperature during the preparation of NDACs on adsorption of Cr^6+^ ions was investigated. The data discovered that the uptake of the Cr^6+^ ions onto NDACs showed a very small increase with temperature increasing from 600 up to 800 °C. The Cr^6+^ ions removal was 98.12, 97.94 and 98.35%, respectively (Fig. 9). From the economic view, the NDAC carbonized at 600 °C was selected as an adsorbent for the removal of Cr^6+^ ions.

#### Influence of pH

**Figure 10:**
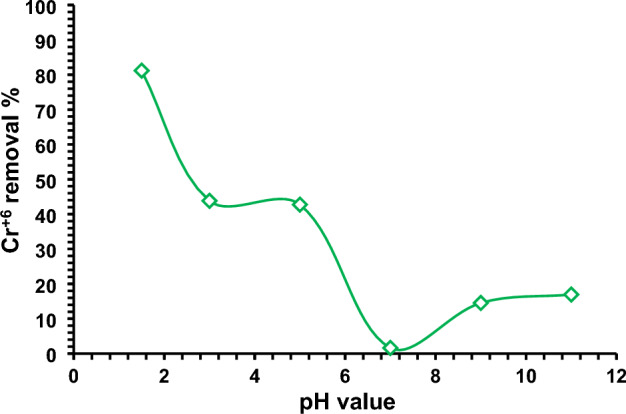
Impact of solution pH on the Cr^+6^ ions removal % using NDAC600 (1.0 g/L) and 100 mg/L initial Cr^+6^ ions concentration at 25 ± 2 °C.

The pH of an aqueous solution is one of the most important factors in the successful the removal of Cr^6+^ ions due to its effects on the number of ions of surface functional groups of the adsorbent (self-doping nitrogen-activated carbon), the solubility and the speciation of the adsorbate during the adsorption process. The influence of pH values on the removal of Cr^6+^ ions onto the surface of NDAC600 was existing in the Fig. 10, which shows that, when the pH value increased from 1.5 to 7, the adsorption efficiency decreased dramatically from 81.18 to 1.61% while at a given pH above 7 to 11, the removal efficiency increased to 16.94%. From the results, we can conclude that the optimum pH was 1.5, and so all other adsorption experiments were done at pH 1.5. The NDAC600 adsorbent has a point of zero charges at pH 8.8, and relying on the pH at pH_PZC_, the NDAC600 had positively charged for solution where pH is smaller than pH_PZC_ and has basic character where pH_PZC_ is greater than 7. Since in an acidic medium, the basic functional groups (C = N, C = C = N, C≡N, and N–H) on the surface of the adsorbent will extensively be protonated and consequently, the NDAC600 surface and a predominant anionic adsorbate species (HCrO_4_)^–^ may be attracted on a complex system. When NADC600 is placed in a solution containing Cr^+6^ ions, the surface of the NADC600 will become positively charged if the pH of the solution is smaller than the pH_PZC_ of the NADC600. In this case, the pH of the solution is 1.5, which is much lower than the pH_PZC_ of carbon, which is 8.8. Therefore, the surface of the carbon will be positively charged. The reason for the positive charge on the carbon surface is due to the presence of hydrogen ions (H^+^) in the acidic solution^4^. At low pH, the concentration of H^+^ ions in the solution is high, and these ions will bind to negatively charged sites on the carbon surface, leaving a net positive charge. The positively charged carbon surface can attract and adsorb negatively charged Cr^+6^ ions from the solution, which can then be reduced to Cr^+3^ ions by the carbon surface^4^. This reduction process is an important mechanism for removing Cr^+6^ ions from contaminated water^4^. Bandara et al.^4^ reported a similar mechanism as they studied Redox mechanisms of conversion of Cr^+6^ to Cr^+3^ by graphene oxide polymer composite. The results suggested that, the Chemical species of Cr^+6^ depends on pH and their concentrations.

Ismael et al.^5^ synthesized a novel cross-linked chitosan (CMBA) material for the adsorption of Cr^6+^ ions from its water solution and the optimum pH was 1.5.

#### Influence of contact time

**Figure 11:**
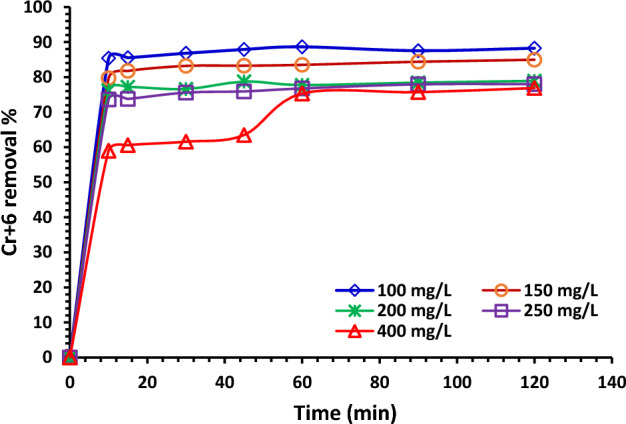
Relation between contact time (min) and Cr^+6^ ions removal % using different initial concentrations on 1.0 g/L of NDAC600 at 25 ± 2 °C, pH 1.5.

Figure 11 shows the impact of the time of contact evolution (10–120 min) on the removal % of Cr^6+^ by using 1.0 g/L of NDAC600 as adsorbent and different initial concentrations of Cr^6+^ (100–400 mg/L). Fortunately, at all the starting concentrations, except 400 mg/L, the removal % is extremely faster, and after just 10 min, the system tends to reach near equilibrium. To ensure equilibrium, the batch experiments were carried out for 120 min. After 10 min at (100, 150, 200 and 250 mg/L) starting concentration of Cr^6+^ ions, the removal % was 85.42, 79.69, 76.21, and 73.62%, while after 120 min, the removal % was 88.24, 84.96,77.99, and 77.99%, respectively. At 400 mg/L starting concentration of Cr^6+^ ions, the removal % became 58.97% after 10 min and increased slowly with increasing agitation time until it reached equilibrium after 60 min and became 76.88% at 120 min^57,58^.

#### Influence of starting concentration

**Figure 12:**
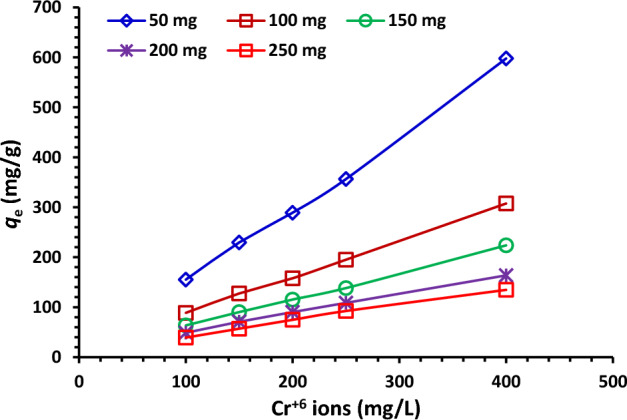
Relation between different starting concentrations (100–400 mg/L) of AB14 dye and *q*_e_ (mg/g) using various NDAC600 doses (0.5 to 2.5 g/L) at 25 ± 2 °C.

The effects of the starting concentrations of Cr^6+^ ions (100–400 mg/L) on the removal % capacity of NDAC600 using various adsorbent doses (0.5 to 2.5 g/L) at 25 ± 2 °C was shown in Fig. 12. At 0.5 g/L adsorbent dose of NDAC600, and at a different starting concentration of Cr^6+^ ions (100, 150, 200, 250 and 400 mg/L), the removal capacity of NDAC600 for Cr^6+^ ions was (155.14, 229.56, 289.04, 356.38 and 597.73 mg/g), respectively. At an adsorbent dose 1.0 g/L of NDAC600, and at different starting concentrations of Cr^6+^ ions (100—400), the adsorption capacity of NDAC600 for Cr^6+^ ions was increased from 88.64 to 304.50 mg/g, while at 1.5 g/L adsorbent dose of NDAC600, and at different starting concentrations of Cr^6+^ ions (100, 150, 200, 250 and 400 mg/L), the adsorption capacity of NDAC600 for Cr^6+^ ions become (63.51, 90.23, 115.26, 138.20 and 223.74 mg/g), respectively. The adsorption capacity of NDAC600 (adsorbent dose = 2.0 and 2.50 g/L) for Cr^6+^ ions at different starting concentrations of Cr^6+^ ions (100–400) increased from 48.79 to 163.65 mg/g and from 39.19 to 134.63 mg/g (Fig. 12). Figure 12 illustrates that the adsorption capacity of NDAC600 for Cr^6+^ ions at equilibrium (*q*_e_) increases as the initial concentration increases. From the results, we deduced that the *q*_e_ at 400 mg/L as starting concentration of Cr^6+^ ions and 0.5 g/L of NDAC600 as adsorbent dosage possessed the highest value, which was 597.728 mg/g. Which is because of the increase in surface area or adsorption sites of the NDAC600. The highest adsorption capacity of NDAC600 for Cr^6+^ ions can be assigned to existence of N and O groups on the NDAC surface which were created after the hydrothermal and pyrolysis processes that act first as strong active sites for the protonation and then attracted with the Cr^6+^ ions predominant anionic species (HCrO_4_)^–^ directly from solution. As mentioned by Bandara et al.^4^, the different chemical species range from CrO4^2−^ at pH above 6 through HCrO_4_^–^ and Cr_2_O_7_^2−^ at pH below 6.5 to H_2_CrO_4_ at pH < 0.7. It is known that when dichromate (Cr2O7^2−^) ions are dissolved in water, they ionize to form chromate (CrO4^2−^) ions and H^+^^4^.

#### Influence of adsorbent dose of NDAC600

**Figure 13:**
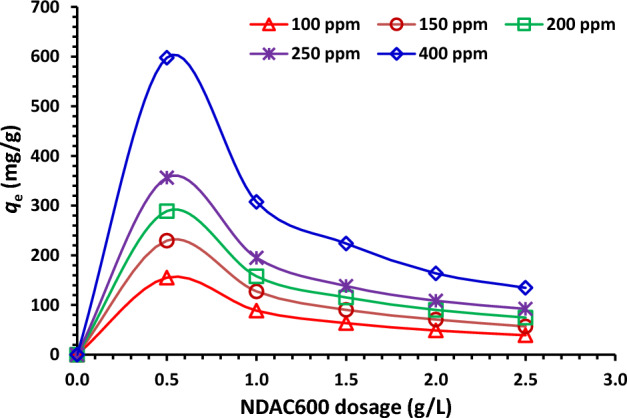
Impact of various adsorbent doses of NDAC600 (0.5 to 2.5 g/L) on the *q*_e_ (mg/g) using various starting concentrations (100–400 mg/L) of Cr^+6^ ions at 25 ± 2 °C.

The relationship between the capacity of adsorption and the adsorbent doses of NDAC600 (0.5, 1.00, 1.5, 2.00, and 2.5 g/L) at various starting concentrations of Cr^6+^ ions (100–400 mg/L) is presented in Fig. 13. as we can see from Fig. 13, the highest adsorption capacities were reached at the lowest adsorbent dose of NDAC600 (0.5 g/L) and increased gradually with increasing the starting concentration of Cr^6+^ ions from 100 to 400 mg/L, they were 155.14, 229.56, 289.04, 356.38, and 597.73 mg/g, respectively. In the contrary, the lowest capacities of adsorption were reached at the highest adsorbent dose of NDAC600 (2.5 g/L) and increased gradually with increasing the starting concentration of Cr^6+^ ions from 100 to 400 mg/L; they were 39.19, 74.68, 74.68, 92.36, and 134.63 mg/g, respectively. This may be ascribed to the fact that, at lower adsorbent doses, the active adsorption sites on the adsorbent surface are efficiently and available for the absorbate while at higher adsorbent doses, a fundamental fraction of reachable adsorption active sites still remains on the adsorbent surface, but the absorbate was already adsorbed from the bulk solution, which lead to decrease in adsorption capacity. The maximum adsorption capacity (*Q*_m_) in this study is higher than that reported by Ismael et al.^5^, where the *Q*_m_ was 149 mg/g.

#### Equilibrium adsorption isotherms

**Figure 14:**
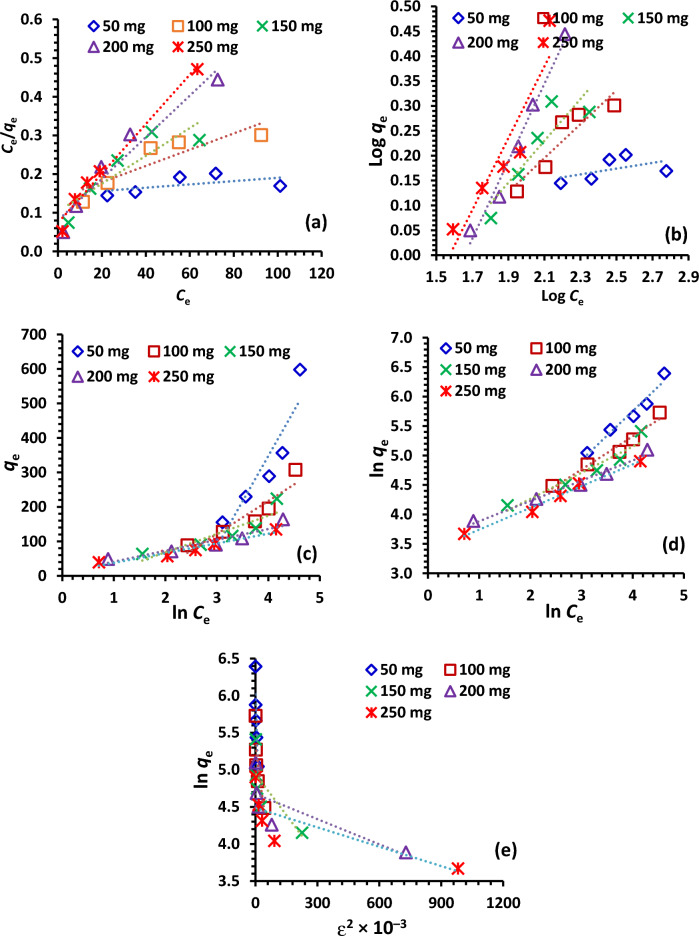
(**a**) Linearized LIM, (**b**) Linearized FIM, (**c**) Linearized TIM, (**d**) Linearized HIM, (**e**) Linearized DRIM of Cr^6+^ ions of 100–400 mg/L initial concentration and 1.0 g/L NDAC600 dose at 25 ± 2 °C.

The equilibrium adsorption data of Cr^6+^ ions on the studied NDAC600 at solution pH 1.5 are interpreted by using LIM, FIM, TIM, DRIM and HIM (Fig. 14a–e). The relevant isotherm parameters of the equilibrium adsorption models were determined by using regression analysis of the experimental data are shown in Table 4. The best-fit model of NDAC600 for Cr^6+^ ions adsorption was Halsey isotherm model due to the high correlation coefficient value (*R*^2^ = 0.9645) than that of Langmuir, Freundlich, Temkin, and Dubinin-Radushkevich isotherm models (Table 4). This means that the model of Halsey isotherm is convenient for the formation of many layers of adsorption at a distance from the surface and the adsorbent has a heterogeneous distribution of pores^58–60^. Based on the adsorption statistical error functions (ASEF) values, the TIM was the best model fits the adsorption data at equilibrium. Worth mentioning, the TIM eliminates the excessively low and high values of the concentrations and presume that, from the value of B and A, which is constant related to heat of sorption (J/mol) and TIM binding constant (L/g), respectively, it could determine the nature of adsorption method. From the values of B and A (Table 5) we can deduce that physical adsorption occurred. These results are in agreement with El-Nemr et al.^35^ who reported that, the adsorption data were defined well by LIM and TIM where the optimum pH value of Acid yellow 36 dye removal was 1.5, with a removal efficiency of 85.86%.

**Table 4:** IM study data of removal of Cr^6+^ ions of various starting concentrations (100–400 mg/L) at 25 ± 2 °C onto 0.5–2.5 g/L NDAC600 doses.

Isotherm model	Isotherm Parameters	NDAC600 (g/L)				
		0.5	1.0	1.5	2.0	2.5
LIM	*Q*_*m*_ (mg/g)	769.23	476.19	277.78	185.19	156.25
	*K*_*a*_ × 10^3^	2.71	15.31	34.85	67.33	86.72
	*R*^*2*^	0.294	0.791	0.777	0.940	0.981
FIM	*1/n*	0.06	0.34	0.42	0.78	0.72
	*K*_*F*_ (mg^1–1/n^L^1/n^g^–1^)	1.07	0.30	0.22	0.05	0.07
	*R*^*2*^	0.271	0.852	0.798	0.978	0.864
HIM	*1/n*_*H*_	0.831	0.563	0.444	0.344	0.370
	*K*_*H*_	18.5	236.0	2010.0	2.300.0	9140.0
	*R*^*2*^	0.960	0.965	0.924	0.981	0.986
TIM	*A*_*T*_	0.07	0.18	0.49	1.35	1.40
	*B*_*T*_	263.17	95.63	53.16	31.59	28.18
	*R*^*2*^	0.848	0.864	0.785	0.893	0.940
DRIM	*Q*_*m*_ (mol kg^–1^)	433.98	208.62	140.43	107.20	88.77
	*K* × 10^6^ (mol kJ^–1^)^2^	96,400	21,300	3700	1100	900
	*E* (kJ mol^–1^)	2.28	4.85	11.62	21.32	23.57
	*R*^*2*^	0.766	0.686	0.583	0.622	0.622

**Table 5:** Error function investigation of the IM of the Cr^+6^ ions removal of various starting concentrations (100–400 mg/L) at 25 ± 2 °C onto 0.5–2.5 g/L NDAC600 doses.

IM	APE%	X^2^	Hybrid	ERRSQ	MPSD	ARE	EABS	RMS
LIM	0.00	0.00	0.01	0.25	0.01	0.00	2.51	0.01
FIM	3.96	3932.10	17,096.1	631,492.6	20.64	3.96	3973.33	19.79
HIM	16.65	13,355.7	58,068.5	411,576.0	86.82	16.65	3207.71	83.27
TIM	6E − 5	0.0	0.0	1.3E − 4	2.9E − 4	6E − 5	5.6E − 2	2.8E − 4
DRIM	0.15	5.65	24.56	907.29	0.78	0.15	150.61	0.75

#### Kinetics model

**Figure 15:**
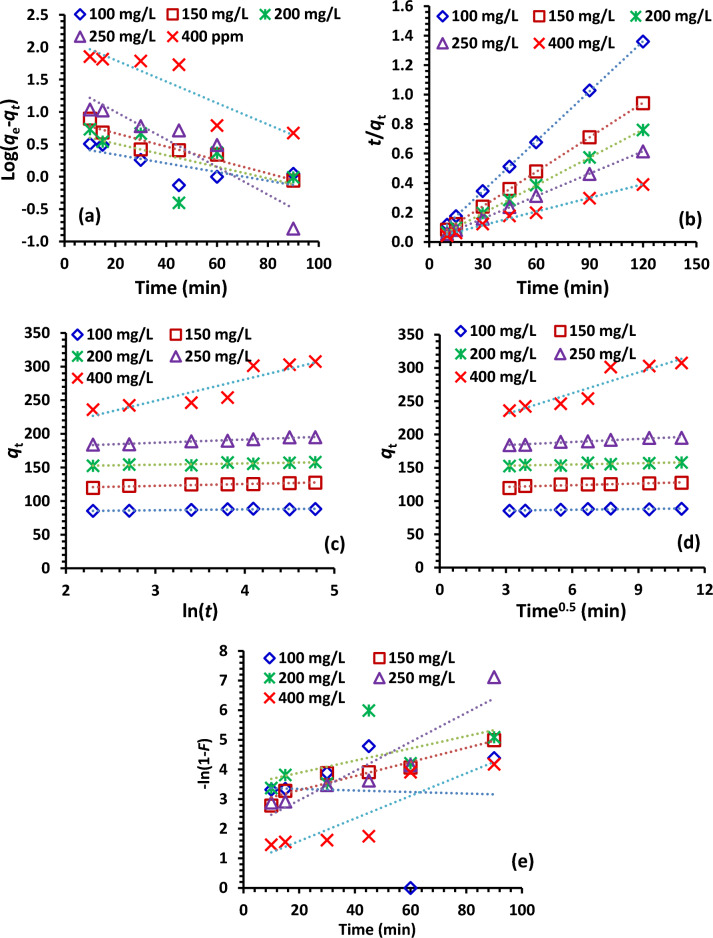
(**a**) PFOM, (**b**) PSOM, (**c**) EM, (**d**) IPDM, (**e**) FDM of removal of Cr^+6^ ions of initial concentration (100–400 mg/L) by NDAC600 (1.0 g/L) at 25 ± 2 °C.

To understand and determine the mechanism that was involved during the adsorption process, the adsorption kinetics models should be studied. The kinetic factors for the five applied kinetics models to fit the experimental data of removal of Cr^6+^ ions at various starting concentrations (100–400 mg/L) onto 0.5–2.5 g/L NDAC600 doses at 25 ± 2 °C are stated in Tables 6 and 7 and in Fig. 15a–e. The five kinetic models namely, pseudo-first-order (PFOM), pseudo-second-order (PSOM), film diffusion (FDM), Elovich (EM) and intraparticle diffusion (IPDM). Based on the evaluated data presented in Table 6 and 7, and both regression correlation coefficient (*R*^2^) and the calculated adsorption capacity (*q*_e_) values, the PSOM has the *R*^2^ near or equal to unity, is the best-fit model. *R*^2^ value of the PSOM is the highest among all the kinetics models explored. The *R*^2^ values were close to unity with all the 100–400 mg/L of initial concentrations and at 0.5–2.5 g/L adsorbent doses. However, this result indicates the applicability of the NDAC600 adsorbent to the removal of Cr^6+^ ions from water. As shown in Tables 6 and 7, the adsorption capacity increased with increasing the starting concentrations from 100 to 400 mg/L and decreased by increasing the NDAC600 doses from 0.5 to 2.5 g/L suggesting a rapid rate of attraction of Cr^6+^ ions onto NDAC600 kinetic process. According to El-Nemr et al.^35^, the adsorption kinetics of Acid yellow 36 dye onto nitrogen doped activated carbon was best described using a PSOM, with R^2^ = 1 indicating that the AY36 dye adsorption mechanism onto NDAC800 was governed by chemisorption.

**Table 6:** PFOM and PSOM rate constants and calculated and experimental *q*_e_ values of removal of Cr^+6^ ions of various starting concentrations (100–400 mg/L) onto 0.5–2.5 g/L NDAC600 doses at 25 ± 2 °C.

Parameter			PFOM			PSOM		
NDAC600 (g/L)	AY36 (mg/L)	*q*_*e*_ (exp.)	*q*_*e*_ (calc.)	*k*_*1*_ × 10^3^	*R*^*2*^	*q*_*e*_ (calc.)	*k*_*2*_ × 10^3^	*R*^*2*^
0.5	100	155.14	13.70	16.58	0.846	156.25	4.137	1.000
	150	229.56	9.03	30.63	0.802	232.56	8.039	1.000
	200	289.04	15.52	42.38	0.985	294.12	6.422	1.000
	250	356.38	412.10	68.17	0.821	384.62	0.298	0.988
	400	597.73	454.15	49.05	0.823	625.00	0.179	0.987
1.0	100	88.64	3.00	15.43	0.575	88.50	29.695	1.000
	150	127.44	7.56	23.72	0.915	128.21	8.947	1.000
	200	157.83	4.89	20.50	0.371	158.73	10.727	1.000
	250	194.99	26.91	49.05	0.869	196.08	4.563	1.000
	400	307.50	135.58	38.23	0.817	322.58	0.467	0.994
1.5	100	63.51	2.39	32.24	0.964	63.69	35.213	1.000
	150	90.22	3.26	31.78	0.948	90.09	35.203	1.000
	200	115.26	9.09	47.67	0.985	116.28	12.752	1.000
	250	138.20	5.77	21.19	0.720	136.99	19.032	1.000
	400	223.74	132.04	35.01	0.845	238.10	0.427	0.989
2.0	100	48.78	1.38	28.10	0.662	49.02	50.140	1.000
	150	70.84	2.43	23.72	0.837	70.92	28.813	1.000
	200	90.15	5.75	24.64	0.971	90.91	11.863	1.000
	250	108.58	9.55	49.74	0.913	108.70	13.435	1.000
	400	163.65	83.52	50.44	0.871	169.49	1.119	0.996
2.5	100	39.19	0.62	29.71	0.988	39.22	127.500	1.000
	150	56.92	2.30	26.48	0.919	57.14	31.901	1.000
	200	74.68	3.43	23.03	0.890	75.19	19.654	1.000
	250	92.36	21.55	66.56	0.867	93.46	8.544	1.000
	400	134.63	53.81	42.14	0.875	140.85	1.393	0.998

**Table 7:** EM, IPDM, and FDM data of the adsorption of Cr^+6^ ions of various initial concentrations (100–400 mg/L) onto 0.5–2.5 g/L NDAC600 doses at 25 ± 2 °C.

NDAC600 (g/L)	AY36 (mg/L)	EM			IPDM			FDM		
		*β*	*α*	*R*^*2*^	*K*_*dif*_	*C*	*R*^*2*^	*K*_*FD*_	C	*R*^*2*^
0.5	100	0.18	1.03E + 11	0.938	1.68	137.07	0.876	0.02	2.43	0.846
	150	0.25	1.03E + 24	0.926	1.19	218.35	0.827	0.03	4.74	0.172
	200	0.25	7.18E + 29	0.958	1.24	276.87	0.897	0.04	2.92	0.985
	250	0.024	1.73E + 03	0.746	13.82	212.93	0.814	0.07	0.15	0.821
	400	0.01	2.77E + 03	0.743	23.28	350.66	0.822	0.05	0.27	0.823
1.0	100	0.79	3.76E + 28	0.802	0.38	84.60	0.701	0.00	3.39	0.002
	150	0.36	2.26E + 18	0.920	0.85	118.69	0.845	0.02	2.82	0.915
	200	0.50	5.19E + 32	0.727	0.63	151.11	0.714	0.02	3.47	0.371
	250	0.21	1.54E + 16	0.976	1.53	179.49	0.960	0.05	1.98	0.869
	400	0.03	3.84E + 03	0.822	10.46	199.16	0.861	0.04	0.82	0.817
1.5	100	1.37	4.52E + 35	0.952	0.23	61.24	0.897	0.03	3.28	0.964
	150	0.93	4.61E + 34	0.841	0.32	87.16	0.720	0.03	2.71	0.159
	200	0.46	1.61E + 21	0.953	0.68	108.72	0.886	0.05	2.54	0.985
	250	0.49	5.07E + 27	0.769	0.61	131.55	0.668	0.02	2.51	0.096
	400	0.04	6.60E + 02	0.821	9.17	124.89	0.904	0.04	0.53	0.845
2.0	100	1.86	1.62E + 37	0.861	0.17	47.11	0.817	0.03	3.57	0.662
	150	1.01	1.45E + 29	0.939	0.30	67.81	0.849	0.02	3.37	0.837
	200	0.54	1.72E + 19	0.987	0.59	84.01	0.958	0.02	2.75	0.971
	250	0.51	1.89E + 22	0.956	0.61	102.57	0.902	0.05	2.43	0.913
	400	0.07	1.22E + 04	0.814	4.64	115.87	0.858	0.05	0.67	0.871
2.5	100	5.38	5.68E + 88	0.983	0.06	38.59	0.953	0.03	4.15	0.988
	150	1.16	5.14E + 26	0.943	0.26	54.29	0.862	0.03	3.21	0.919
	200	0.75	2.60E + 22	0.942	0.41	70.51	0.863	0.02	3.08	0.890
	250	0.38	5.17E + 13	0.943	0.83	84.07	0.929	0.07	1.46	0.867
	400	0.08	6.37E + 03	0.879	3.95	94.54	0.896	0.04	0.92	0.875

### Adsorption mechanism of Cr^6+^ ions by NDAC600

**Scheme 1:**
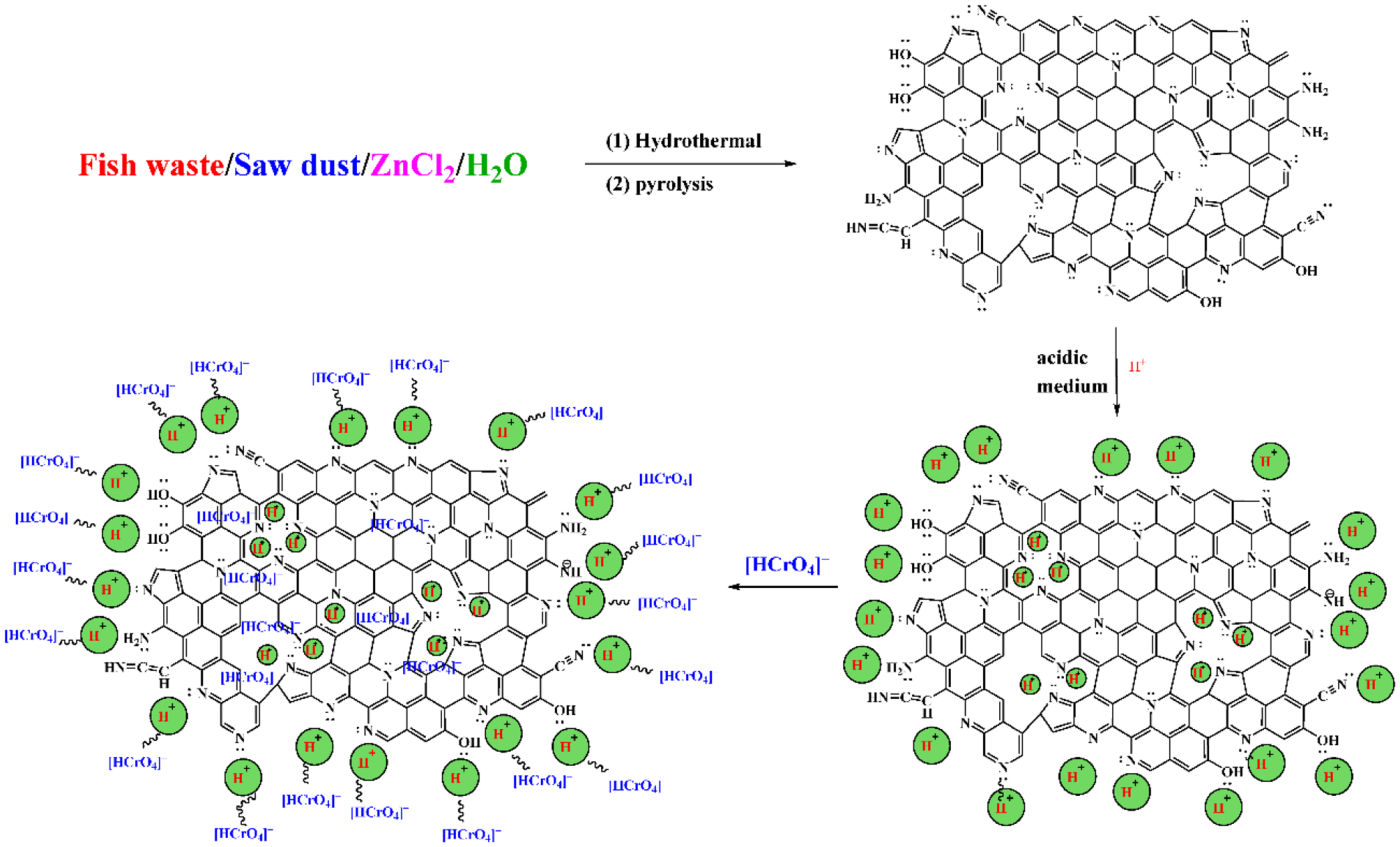
Probable mechanism for the Cr^6+^ ions adsorption of onto the NDAC600.

The probable mechanism for the removal of the Cr^6+^ ions onto NDAC600 was explained in Scheme 1. After the pyrolysis of the hydrothermal product of (Fish waste/ZnCl_2_/sawdust in water) at 600 °C, many functional groups were formed on the adsorbent (NDAC600) surface like allene C = C = C, ketamine C = C = N, amide N–H, hydroxyl O–H, C-N and isonitrile cyano C≡N groups. The mechanism of the removal of Cr^6+^ ions in an acidic medium may be achieved via physical interaction due to electrostatic interaction between the positive hydrogen ions in the bulk solution and the nitrogen and oxygen functional groups on the NDAC600 surface, then surface charge became positive; subsequently electrostatic interaction was occurred between the positively charged surface and the predominant chromium anionic species (HCrO_4_)^–^.

### Comparison results of *Q*_m_ of Cr^6+^ compared to those found in literature

The *Q*_m_ of Cr^6+^ ions and other pollutants removal using different N-doped activated carbon summarised in the literature were compared to the NDAC600 adsorbent (Table 8). This proved that NDAC600 was adequate for removing Cr^6+^ ions from water. The NDAC600 shows *Q*_m_ (769.23 mg/g), comparable to those mentioned in Table 8 for different pollutants adsorption. İt was noticed that the NDAC600 was more effective than other N-doped biochar for the adsorption of Cr^+6^ ions and other pollutants.

**Table 8:** Comparison of* Q*_m_ of Cr^**6+**^ and other pollutants by different N-doped activated carbon.

Materials	Pollutant	*Q*_m_ (mg g^−1^)	Ref
NDAC600	Cr^6+^	769.23	This work
Sawdust N-doped AC	Cr^6+^	14.97	^61^
N-doped multi walled carbon nanotubes	Cr^6+^	35.26	^62^
Chitosan-based N-doped AC	Cr^6+^	20.04	^63^
N-doped magnetic porous carbon	Cr^6+^	16	^64^
N-GAC 400 °C	Cr^6+^	15.15	^29^
N-doped Medulla tetrapanacis biochar	Congo red	714.29	^65^
*Phragmites Australis N*-doped Biochars	Acid red 18	134.17	^66^
Leather solid wastes N-doped activated carbons	Phenols	282	^67^
Leather solid wastes N-doped activated carbons	Phenols	73	^67^
Alginate/chitosan film M4	Congo red	555.55	^3^
Alginate/chitosan film M4	Coralene dark Red2B	434.78	^3^
Alginate/chitosan film M5	Congo red	625	^3^
Alginate/chitosan film M5	Coralene dark Red2B	370.37	^3^
Carbonized peanut shell	Methylene blue	5.34	^68^
Carbonized chestnut shell	Methylene blue	5.13	^69^

The Cr^6+^ species in solution are expected to interact with NDAC600 through amines (–NH_2_), and hydroxyl (–OH) (Fig. 8). In the currently investigated pH, the amines in the NDAC600 will follow the following equation at acidic pH:

22 $${\text{R}} - {\text{NH}}_{{2}} + {\text{H}}^{ + } {\text{R}} - {\text{NH}}_{{3}}^{ + } ,$$

which suggests that the –NH_2_ group in NDAC600 will remain positively charged^4^. Therefore, it can be theorized that the hydroxyl and amine protonated groups in NDAC600 are responsible for the removal of Cr^6+^ species from the aqueous solution by forming electrostatic attractions with the negatively charged Cr^6+^ species^4^ thereby, removing them from the solution. However, HCrO_4_^–^ would bind to positively charged functional groups on the beads according to the equations below:

23 $${\text{R}} - {\text{OH }} + {\text{ HCrO}}_{{4}}^{-} \to {\text{R}} - {\text{OH}}^{......} {\text{HCrO}}_{{4}}^{-}$$

24 $${\text{R}} - {\text{NH}}_{{3}}^{ + } + {\text{ HCrO}}_{{4}}^{-} \to {\text{R}} - {\text{NH}}_{{3}}^{ + ......} {\text{HCrO}}_{{4}}^{-}$$

## Conclusion

This work reports an efficient, eco-friendly, cheap and simple method for preparing novel self-NDACs at various temperatures 600, 700, and 800 °C as carbon-based materials produced via a hydrothermal method. NDAC at 600 °C was used to remove Cr^6+^ ions from water effectively. The NDACs adsorbents possessed multifunctional active sites within a high surface area of 437.51–680.86 m^2^/g and a microporous structure of 1.8305–2.0133 nm. The NDAC600 adsorbent exhibits a *Q*_m_ for Cr^6+^ uptake of 769.32 mg/g at pH 1.5. At all the starting concentrations (100, 150, 200 and 250 mg/L) expect 400 mg/L, the removal efficiency % is extremely faster and achieved within 10 min. Obviously, the adsorption process was also combined with rapid kinetics where the equilibrium time required for the 85.42% removal of Cr^6+^ ions for 100 mg/L starting concentration onto 1.0 g/L of NDAC600 was 10 min. Based on the value of the *R*^2^ = 0.965, the best fit model of NDAC600 for Cr^6+^ ions adsorption was the Halsey isotherm, and according to functional error analysis, the Temkin isotherm is the best-fit with the equilibrium adsorption results. The PSOM, which possessed a *R*^2^ = 1, is the best-fit kinetic model. These results suggested that there was a rapid rate of attraction of Cr^6+^ ions onto NDAC600, and the physical adsorption occurred with the formation of several layers of adsorption at a distance from the non-homogeneous surface of the adsorbent.

## Data Availability

The datasets used in this investigation are accessible for review upon request from the corresponding author of the paper.
